# Emergence as an outbreak of the HIV-1 CRF19_cpx variant in treatment-naïve patients in southern Spain

**DOI:** 10.1371/journal.pone.0190544

**Published:** 2018-01-08

**Authors:** Carmen M. González-Domenech, Isabel Viciana, Luis Delaye, María Luisa Mayorga, Rosario Palacios, Javier de la Torre, Francisco Jarilla, Manuel Castaño, Alfonso del Arco, Encarnación Clavijo, Jesús Santos

**Affiliations:** 1 Biomedical Research Institute of Malaga (IBIMA), Málaga, Spain; 2 Infectious Diseases and Clinical Microbiology Unit, Virgen de la Victoria Hospital, Málaga, Spain; 3 Departamento de Ingeniería Genética, CINVESTAV. Guanajuato, Irapuato, México; 4 Infectious Diseases and Clinical Microbiology Unit, Carlos Haya Regional Hospital, Málaga, Spain; 5 Infectious Diseases Unit, Service of Internal Medicine, Costa del Sol Hospital, Marbella, Spain; 6 Service of Internal Medicine, Antequera Local Hospital, Málaga, Spain; University of Cincinnati College of Medicine, UNITED STATES

## Abstract

**Background:**

CRF19_cpx is a complex circulating recombination form (CRF) of HIV-1. We describe the characteristics of an outbreak of the CRF19_cpx variant among treatment-naïve patients in southern Spain.

**Methods:**

The study was undertaken at the Virgen de la Victoria Hospital, a reference centre for the analysis of HIV-1 genotype in Malaga (Spain). Subtyping was performed through REGA v3.0 and the relationship of our CRF19_cpx sequences, among themselves and regarding other reference sequences from the same variant, was defined by phylogenetic analysis. We used PhyML program to perform a reconstruction of the phylogeny by Maximum Likelihood method as well as further confirmation of the transmission clusters by Bayesian inference. Additionally, we collected demographic, clinical and immunovirological data.

**Results:**

Between 2011 and 2016, we detected 57 treatment-naïve patients with the CRF19_cpx variant. Of these, 55 conformed a very well-defined transmission cluster, phylogenetically close to CRF19_cpx sequences from the United Kingdom. The origin of this subtype in Malaga was dated between 2007 and 2010. Over 50% of the patients presented the non-nucleoside reverse transcriptase inhibitor G190A resistance mutation. This variant was mostly represented by young adult Spanish men who had sex with men. Almost half of them were recent seroconverters, though a similar percentage was diagnosed at a late state of HIV infection. Five cases of AIDS and one non-AIDS defined death occurred during follow-up. The majority of patients treated with first-line combination antiretroviral therapy (ART) responded.

**Conclusions:**

We report the largest HIV-1 CRF19_cpx cohort of treatment-naïve patients outside Cuba, almost all emerging as an outbreak in the South of Spain. Half the cases had the G190A resistance mutation. Unlike previous studies, the variant from Malaga seems less pathogenic, with few AIDS events and an excellent response to ART.

## Introduction

CRF19_cpx is a complex circulating recombination form (CRF) of HIV-1, exhibiting a mosaic structure with multiple segments of subtypes A, D, and G. Although molecular epidemiology studies suggest a central African origin for this variant [1–2], CRF19_cpx was first described in HIV-1 patients from Cuba [1], where it was imported around the mid-1980s and rapidly disseminated within local transmission networks [2]. This recombinant form now represents almost 4% of all subtypes and more than 17% of all the CRFs in newly diagnosed Cuban patients [3–5]. Other genetic variability studies show even higher prevalence values, reaching up to ~28% in Cuba [6]. Additionally, examination of the Los Alamos HIV Database (LANL, http://www.hiv.lanl.gov/content/sequence/HIV/mainpage.html) shows that most of the entries for CRF19_cpx also come from Cuba (217 out of 285 sequences, last accessed in January 2017).

Clinically, patients with the subtype CRF19_cpx are mostly linked to a rapid progression to AIDS [7]. CRF19_cpx has also been found as one of the most prevalent viral variants, with multiple drug resistance mutations in HIV-1 therapy-naïve patients in Cuba [5, 8]. Consequently, the subtype CRF19_cpx seems to be associated with greater pathogenicity and common resistance mutations [7, 9].

As mentioned, and despite the very low spread of the CRF19_cpx variant outside Cuba, the existence of a few cases of this recombinant form was recently reported in Tunisia and Spain [10–11]. The United Kingdom, Greece and France are also represented, with certain sequences in the LANL (48, 2 and 1 sequences, respectively; last accessed in January 2017).

Here, we present a major expansion of this HIV-1 CRF outside Cuba. The variant has been transmitted in southern Spain as an outbreak among MSM, diagnosed between 2011 and 2016.

## Methods

### Study population and subtype assignment

The study was undertaken at the Virgen de la Victoria Hospital, a reference centre of the study of HIV-1 genotype drug resistance for six hospitals from the region of Malaga (southern Spain). A genotype resistance test has been routinely undertaken in our centre since 2004 for all the patients with confirmed HIV-1 infection at the time of diagnosis and before starting combination antiretroviral therapy (ART). A partial region of HIV-1 *pol* gene, encoding the complete protease (PR) and partial reverse transcriptase (RT), was sequenced using RT–PCR and Sanger sequencing (Trugene HIV Genotyping Kit®, Siemens Healthcare Diagnostics Inc., Tarrytown, NY, USA) or 454 pyrosequencing (GS Junior Titanium Sequencing Kit® Roche Diagnostics Gmbh, Mannheim, Germany), depending on the date of sample collection (before or after 2014, respectively). The subtype for each FASTA sequence provided was assigned through REGA v3.0 and sequences determined as CRF19_cpx subtype, afterwards confirmed by phylogenetic analysis.

### Phylogenetic analysis

The relationship of our CRF19 cpx sequences among themselves and with regards to the epidemic of this subtype worldwide was characterized by means of a phylogenetic analysis with another 254 reference sequences of the same variant retrieved from the LANL (198 sequences from Cuba, 47 from the United Kingdom, 4 from Spain, 3 from the USA, 1 from Greece and another one from Tunisia). The PR and RT sequences (average overall length of 913 nt) were aligned by ClustalX. The phylogenetic reconstruction was inferred by maximum likelihood method with PhyML v3.0 program [12–13]. The cluster reliability was supported by two non-parametric branch-supports implemented in the mentioned software: bootstrapping with 100 replications and SH-like aLRT test, another branch-support measure in line with the SH tree-selection method [14]. The best substitution model was determined with the Findmodel option included in MEGA v6.0 [15], assuming the lowest AIC (Akaike Information Criterion) score as the selection criterion [16].

The phylogeny inferred was confirmed by a Bayesian approach using MrBayes v3.2 program [17] and visualized by the graphical viewer FigTree (http://tree.bio.ed.ac.uk/software/figtree/).

### Dating the most recent common ancestor (tMRCA) of the variant in our area

An outbreak cluster was identified in our cohort and extracted from the tree and time-resolved in BEAST. For this purpose, we applied a Coalescent-based Bayesian Markov Chain Monte Carlo (MCMC) approach with the program BEAST v2 [18]. Under a relaxed uncorrelated lognormal clock, we set the GTR G+I substitution model and a prior lognormal distribution of 5x10^-3^ substitutions *per* site *per* year (standard deviation = 0.5) in the evolution rate, as the general heterogeneity rate reported in the literature for all subtypes in the *pol* gene [19–20]. We chose the most appropriate coalescent model (Bayesian and Extended Bayesian Skylines, constant or exponential) to infer the population dynamics of this outbreak, based on the lowest value of Akaike’s Information Criterion (AICM). The MCMC was run as default with chain lengths of 100 million states, sampling estimates every 1000^th^ generation. All the parameters were estimated using the software Tracer 1.6 (http://tree.bio.ed.ac.uk/software/tracer/), accepting only traces with an effective sample size (ESS) of >200 to assess the tMRCA.

### Description and statistical analyses of the study population characteristics

Additionally, we collected demographic, clinical and immunovirological data as well as therapy-related information for the patients with the CRF19 variant. All the patients signed an Informed Consent at their first visit to each hospital, containing explicit agreement to use the routine data under confidentiality and anonymized, as performed here. We carried out a statistical analysis of these variables with the software SPSS 16.0. Prior to the descriptive analysis, we studied the distribution of the corresponding variable in the whole cohort using the mean or the median, according to whether it adjusted or not to normality, respectively. Comparison of proportions was performed by the bilateral Fisher test. For quantitative variables following a non-normal distribution, the Wilcoxon non-parametric test was used. To evaluate the degree of association or independence of quantitative variables (normally distributed) with a dichotomous category, the means were compared in the two categories with the Student *t* test. In all cases statistical significance was set at *p*< 0.05.

### Drug resistance

The resistance mutations were predicted using Stanford algorithm v7.1.1, available in the Stanford University HIV Drug Resistance Database (https://hivdb.stanford.edu/hivdb/by-mutations/) [21].

### Nucleotide sequence accession numbers

The sequences were made publicly available in GenBank (http://www.ncbi.nlm.nih.gov/genbank), under accession numbers **KY558407**-**KY558462** and **KY766062** for PR genes, and **KY558463**-**KY558518** and **KY766063** for RT genes.

## Results

**Fig 1:**
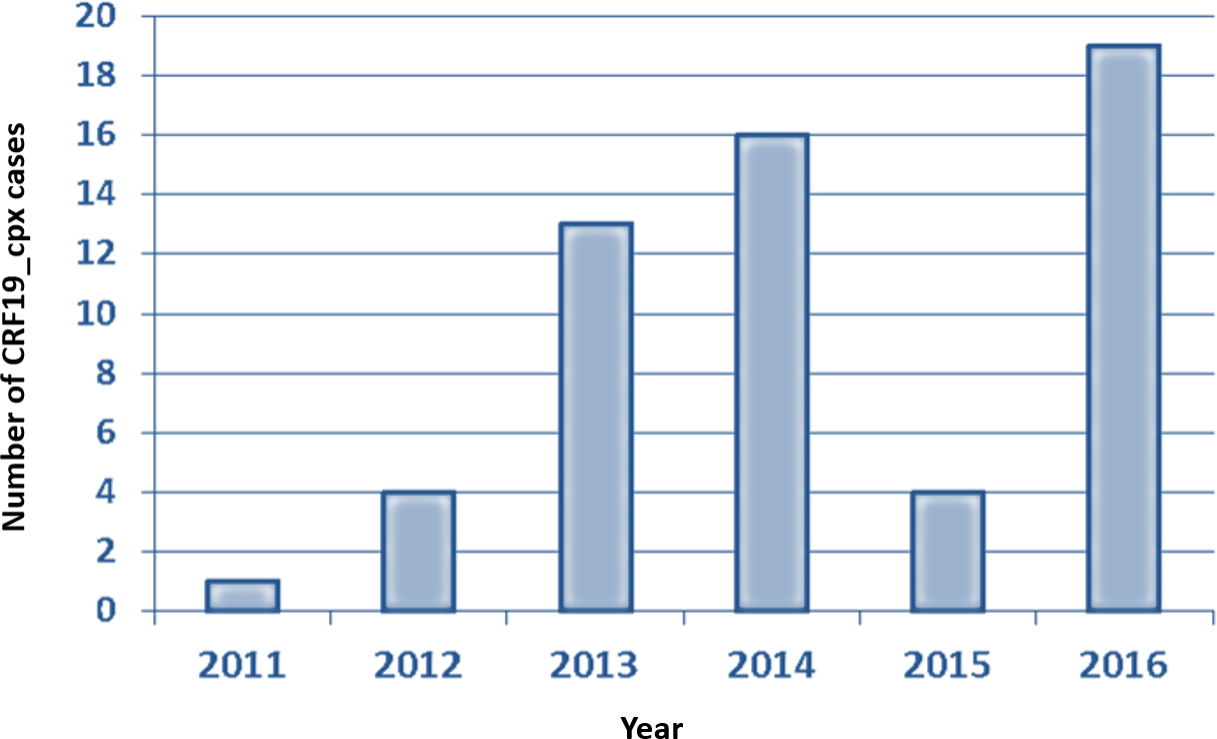
Cases of CRF 19_cpx subtype over time. Number of CRF19_cpx variants detected in the Hospital Virgen de la Victoria, from the first case found until the end of the study period (2011–2016).

We subtyped all sequences provided by any kind of genotype test performed in our hospital from treatment-naïve patients diagnosed since 2004, finding the first case of the CRF19_cpx variant in 2011 (**Fig 1**). From January 2011 to December 2016, a total of 2566 resistance studies were carried out in naïve patients; 57 (2.2%) had sequences consigned in REGA as subtype CRF19_cpx or similar [Recombinant of 19_cpx, B and Subtype D (19_cpx)] (results in **S1 Table**). The highest prevalence for this subtype was found in 2016, with 19 patients out of 438 (4.3%).

**Fig 2:**
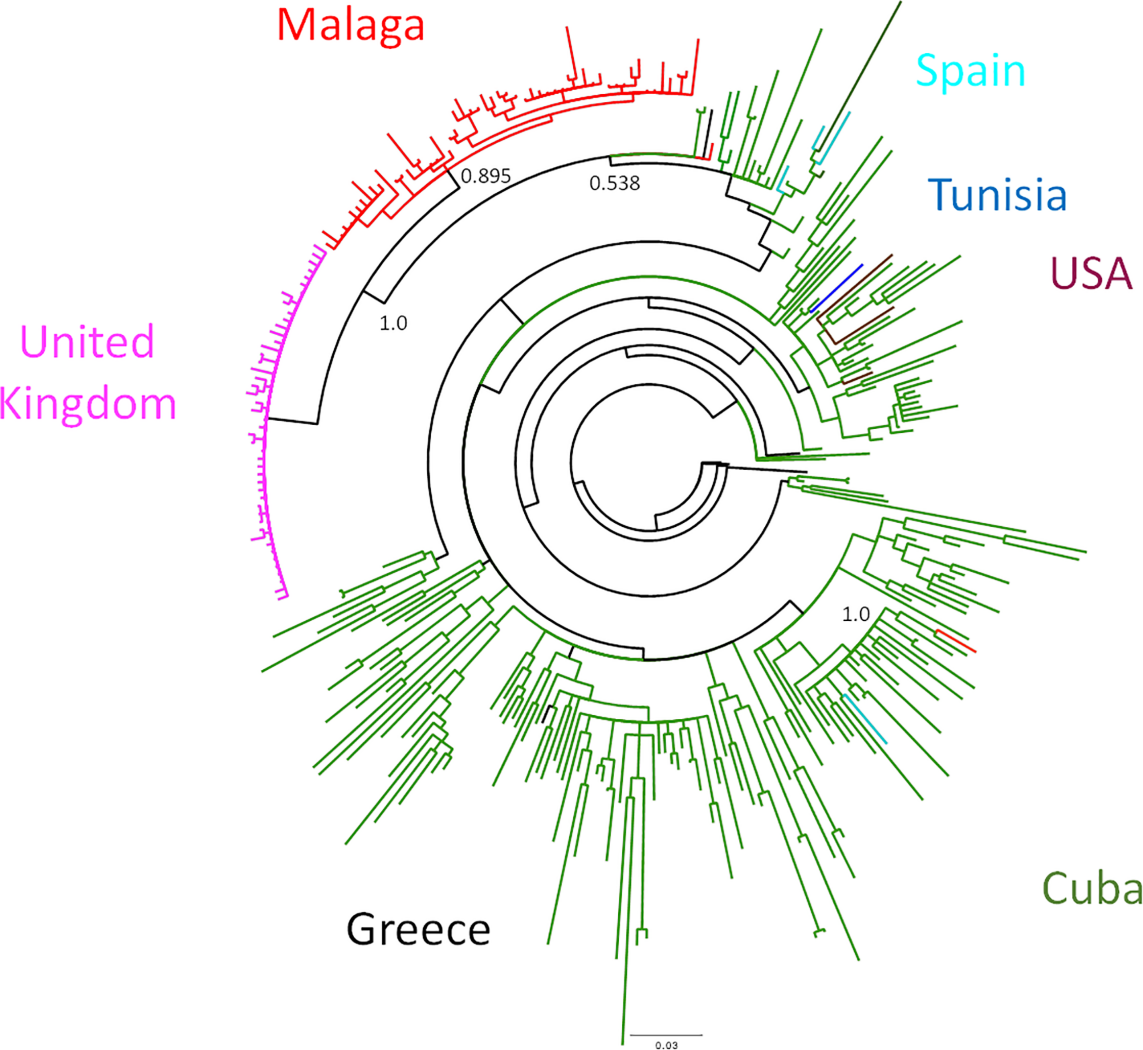
Bayesian maximum clade credibility phylogenetic tree inferred by MrBayes v3.2 program showing our CRF19_cpx sequences and another 254 reference sequences from the same variant retrieved from LANL. Each patient from our cohort is represented in red by their sample ID, while reference sequences appear in different colours according to the country of sampling (green: Cuba; pink: United Kingdom; turquoise: Spain (other than our cohort); brown: USA; black: Greece; blue: Tunisia). This figure is depicted in detail as a supplementary material (**S2 Fig**).

**Fig 3:**
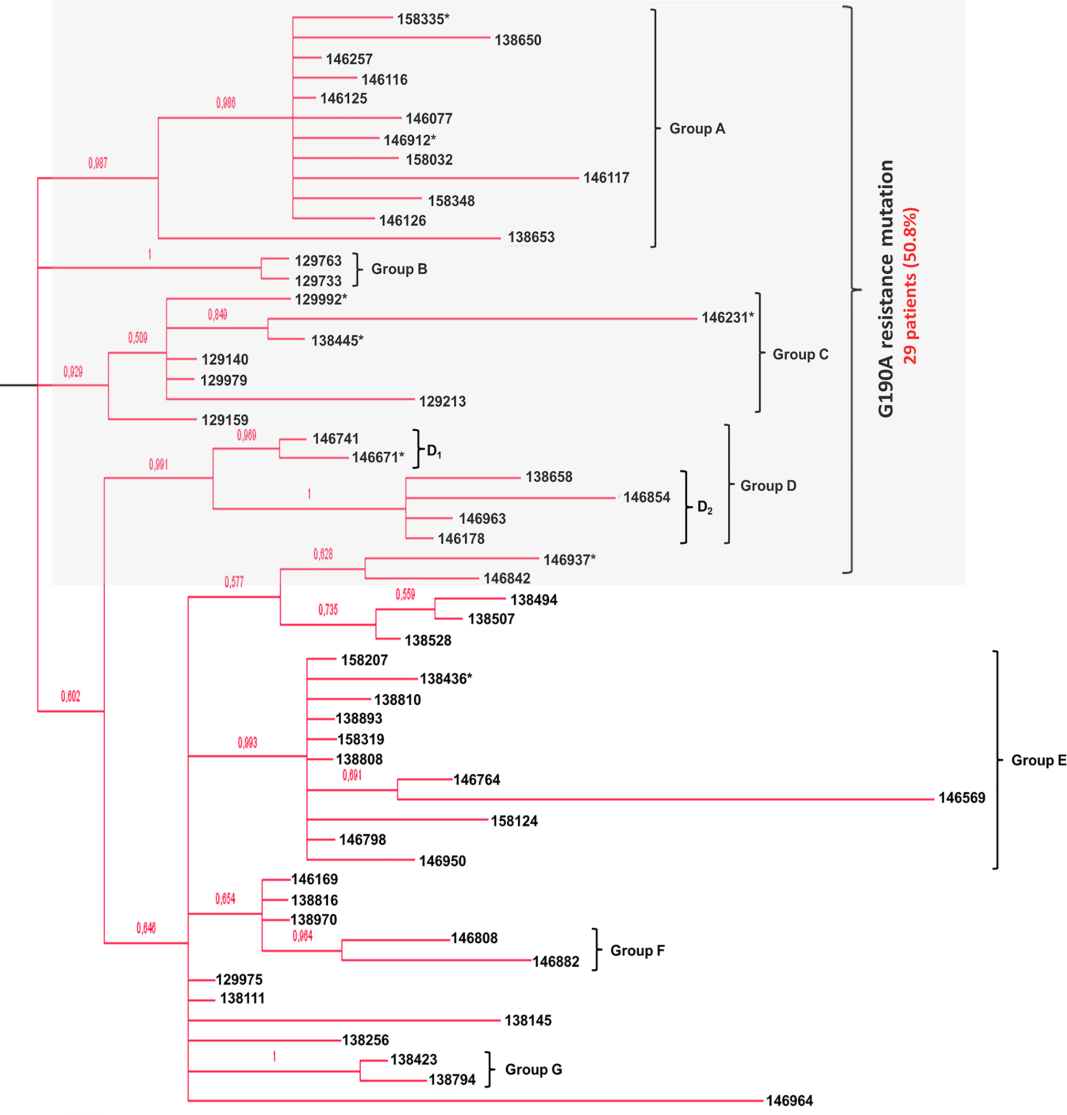
Subtree with the 55 CRF19 cpx sequences grouped together (pp = 0.895) conforming the identified outbreak as depicted in Bayesian inference of the phylogeny (see Fig 2). Sequences presenting the G190A mutation are highlighted within the light grey shaded square. Asterisks indicate the detection of V179I/A as applicable.

The Bayesian approach confirmed the presence of a local transmission cluster, with an associated posterior probability (pp) of 0.895 (**Fig 2**, shown in detail in **S2 Fig**). We inferred the phylogeny with the GTR model using a Gamma distribution (+G) with 5 categories and assuming a fraction of sites as evolutionarily invariant positions (+I), according to results from FindModel (shown in **S1 Fig**). Almost all of the sequences studied (55 out of 57) appeared phylogenetically close (pp = 1; bootstrap = 76%; and SH-like aLRT = 95%) to another 47 CRF19_cpx reference sequences sampled in the United Kingdom (UK) between 2008 and 2010 (**Fig 2, S3A** and **S4A Figs**). These 55 study sequences, range sampled from 2011 to 2016, conformed a well-defined monophyletic cluster in the ML tree as well (bootstrap = 55%; SH-like aLRT = 89%), not broken down by adding any of the references from LANL or even those mentioned from the UK (**S3 and S4 Figs**). In addition, the Bayesian inference of the phylogeny showed that 42 sequences from patients within this outbreak (76.3%) were comprised in seven subclusters of different size (pp≥0.9) (**Fig 3, Table 1**). All these subclusters had also correspondence in the ML tree, with one or both of the branch-support measures and thresholds considered (**S3B and S4B Figs**). Finally, the most recent common ancestor (MRCA) of this outbreak was dated at 2009 (2007.5–2010.0, 95% HPD) by the Bayesian skyline growth with a lognormal relaxed molecular clock, selected as reporting the lowest AICM value (**S2 Table**).

**Table 1:** Summary of the main demographic, clinical and virological data for the seven subclusters found (pp ≥0.9), named according to appearance in the phylogeny inferred by Bayesian analysis (Fig 2, S2 Fig).

		Cluster A	Cluster B	Cluster C	Cluster D	Cluster E	Cluster F	Cluster G
**N° patients**		12	2	7	6	11	2	2
**Sampling period**		2014–2016	2012	2011–2014	2014–2016	2013–2016	2016	2013, 2014
**Origin**		Spain	Spain, Argentina	Spain, Argentina	Spain	Spain	Spain	Spain
**Risk behaviour**	**MSM**	12	2	6	5	9	2	2
	**HTX**	0	0	0	1	0	0	0
	**Others** **/unknown**	0	0	1	0	2	0	0
**Initial viral load** **(log copies/mL)**		4.7 (4.4–5.2)	4.4 (4.1–4.8)	4.8 (4.4–5.2)	4.4 (4.2–4.8)	5.2 (4.8–5.6)	5.9 (5.8–6.1)	4.8 (4.7–4.9)
**Initial lymphocyte CD4 count (cells/μL)**		471 (302–637)	513 (419–606)	464 (372–512)	325 (244–323)	317 (257–420)	295 (235–354)	377 (269–485)
**Initial lymphocyte CD8 count (cells/μL)**		1091 (719–1389)	914 (791–1037)	887 (635–984)	860 (652–1029)	992 (502–1368)	1007 (885–1130)	675 (473–878)
**AIDS cases**		0	0	1	1	2	0	0
**G190A mutation**		Yes	Yes	Yes	Yes	No	No	No

On the other hand, there were two sequences phylogenetically separated from each other and from the rest. One of them was sampled in 2013 and showed a close phylogenetic relation (pp = 1.0; bootstrap = 84%; SH-like aLRT = 98%) to reference sequences from Cuba exclusively. The other one, however, sampled in 2016, did not belong to any subgroup (either reference or local clusters) with a high enough confidence value (pp = 0.538, **Fig 2**). Thus, at least three separate routes of introduction the CRF19_cpx HIV-1 occurred in our area. This pattern was robustly confirmed with the two phylogenetic reconstructions obtained, ML tree (also supported by both non-parametric measures) and Bayesian inference.

Regarding antiretroviral drug resistance, we found the G190A mutation associated with different levels of non-nucleoside reverse transcriptase inhibitor (NNRTI) resistance in 29 out of 57 patients with the subtype CRF19_cpx (50.9%), including all the patients from clusters A to D (**Fig 3**). Outside these clusters but within the outbreak, there are 2 more patients with this mutation. In addition, we also detected in 8 patients (14.0%) the polymorphic mutation V179I/A, though this possesses little direct effect on NNRTI susceptibility. Unlike G190A mutation clusters, the patients with the V179 polymorphism did not show any clear grouping with each other, as depicted in **Fig 3** as well as **S3 and S4 Figs** in Supplementary material.

Furthermore, as shown in **Table 2**, 54 out of 57 patients were self-reported men who had sex with men (MSM) (94.7%). All were Spanish, except two patients from Argentina and one from France. The average age of the cohort was 35.7 years (27.2–42.0). The initial CD4 count was 387 cells/μL (259–468). Eight patients (14.0%) had <200 cells/μL at diagnosis and 26 (45.6%) presented a late diagnosis (initial CD4 count <350 cells/μL). In addition, the average first viral load was 4.9 Log_10_ copies/mL (4.3–5.5), with a zenith value of 5.0 Log_10_ copies/mL (4.5–5.5), this latter being lower in patients with the G190A mutation (4.7 *vs*. 5.2, *p* = 0.03). On the other hand, five cases of AIDS (8.8%) were recorded at diagnosis. Only one death was recorded during the study period, due to acute myocardial infarction in a patient two years after his diagnosis and without having any AIDS event during the follow-up period. Finally, 54 patients were being treated with first-line combination ART at the end of the study period, 92.6% of them with viral suppression. Other demographic and clinical data about our cohort as well as the detailed comparison between the groups with and without the G190A mutation are depicted in **Table 2**.

**Table 2:** Characteristics of the 57 patients with the CRF19_cpx variant and comparison of the groups with and without the G190A mutation.

Characteristics of the patients with the CRF19_cpx variant		CRF19_cpx variant with the G190A mutation	CRF19_cpx variant without the G190A mutation	*p-value*	Cohort
**Number of patients**		29 (50.9)	28 (49.1)		57
**Age (years)**		35.9 (27.6–39.9)	35.5 (27.1–42.1)	0.8	35.7 (27.2–42.0)
**Age at diagnosis (years)**		33.4 (26.3–38.5)	33.2 (25.1–39.5)	0.9	33.3 (25.5–39.4)
**Risk behaviour**	**MSM**	27 (93.1)	27 (96.4)	0.6	54 (94.7)
	**HTX**	1 (3.4)	0		1 (1.8)
	**Others/unknown**	1 (3.4)	1 (3.5)		2 (3.5)
**Education**	**No studies/primary school**	3 (10.3)	2 (7.1)	0.2	5 (8.7)
	**Undergraduate**	15 (51.7)	9 (32.1)		24 (42.1)
	**University**	7 (24.1)	8 (28.6)		15 (26.3)
	**Unknown**	4 (13.8)	9 (32.1)		13 (22.8)
**Origin**	**Spain**	27 (93.1)	27 (96.4)	0.2	54 (94.7)
	**Argentina**	2 (6.9)	0		2 (3.5)
	**France**	0	1 (3.6)		1 (1.7)
**Seroconversion time (months)**		17.6 (13.2–44.8)	16.2 (10.3–18.4)	0.07	15.8 (11.4–34.5)
**Initial viral load (log copies/mL)**		4.7 (4.3–5.2)	5.1 (4.5–5.6)	0.07	4.9 (4.3–5.5)
**Initial lymphocyte CD4 count (cells/μL)**		416 (278–570)	358 (248–449)	0.2	387 (259–468)
**Initial lymphocyte CD8 count (cells/μL)**		933 (569–1211)	1153 (741–1648)	0.1	1041 (612–1365)
**Nadir lymphocyte CD4 count (cells/μL)**		369 (292–438)	327 (189–404)	0.1	346 (252–421)
**Zenith viral load (log copies/mL)**		4.7 (4.4–5.2)	5.2 (4.6–5.6)	0.03	5.0 (4.5–5.5)
**Final lymphocyte CD4 count (cells/μL)**		762 (512–940)	696 (526–847)	0.4	729 (518–871)
**Final lymphocyte CD8 count (cells/μL)**		934 (694–1309)	1107 (957–1268)	0.2	1114 (757–1303)
AIDS cases*		3 (10.3)	2 (7.1)	0.5	5 (8.8)
**Death**		1 (3.4)	0	0.3	1 (1.7)
Viral suppression**		24 (88.8)	26 (96.3)	0.4	50 (92.6)

The quantitative variables are expressed as average or median and IQR, and the qualitative variables as n (%).

MSM: Men who have sex with men; HTX: heterosexual transmission.

*Two cases of Kaposi Sarcoma, one oesophageal candidiasis and two *Pneumocystis jirovecii* pneumonia.

**Patients receiving antiretroviral therapy: 27 in both groups, 54 out 57 patients in total.

## Discussion

As far as we know, this study reports the largest cohort of HIV-1 CRF19_cpx outside Cuba. Although the spread of this recombinant outside Cuba has been previously described [10–11], it is the first time arising as an outbreak of such size. CRF19_cpx has been sampled in our area since 2011, with an estimated introduction in 2009. Up to the end of the study period, we detected this subtype in 57 treatment-naïve patients, 55 of them phylogenetically grouped in a well-defined cluster. Phylogenetic analysis showed the proximity to reference sequences of the CRF19_cpx subtype sampled in the United Kingdom between 2008 and 2010. On the other hand, there were also two more sequences of this variant, separate from each other and from the other patients in our area. One of them is not linked to any transmission cluster, not even with other CRF19_cpx references from the LANL. Thus, the pattern shows at least three separate introductions of the CRF19_cpx HIV-1 in the Malaga area, only one of them emerging as an outbreak, with a sharp increase of cases during the study period. All the phylogenetic approaches performed, in addition to the epidemiological data discussed below, support its status of a real outbreak.

The centre of this outbreak is Malaga, with no relation to any other CRF19_cpx sequence from treatment-naïve patients outside this area [22]. The prevalence for this variant constitutes the second highest among non-B subtypes and recombinants in the Costa del Sol area [23]. Moreover, the prevalence in 2016, the last year included in the study, was over 4%, a figure very similar to that observed in Cuba itself [3]. Kouri *et al*. highlighted the high fitness score of the CRF19_cpx subtype in the PR region [7]. Although we did not determine the replicative capacity of the specific strains of this variant circulating in our area, its persistence for a long time and its increasing transmission support a high viral fitness.

CRF19_cpx has also been associated with multiple drug resistance mutations in therapy-naïve Cuban patients [5, 8]. In this respect, more than half of our cohort possessed the G190A mutation associated with different levels of NNRTI resistance (intermediate resistance for efavirenz, potential low level resistance for etravirine, high level resistance for nevirapine, and low level resistance for rilpivirine). However, the high prevalence of this specific drug resistance differs strikingly from other studies, where it is seldom found at diagnosis [5, 24]. Indeed, we detected the polymorphism at position 179 of the RT gene with a lower frequency than seen in another Spanish cohort of 9 CRF19_cpx patients [11]. Unlike the G190A mutation though, the above mentioned polymorphism only confers low-level resistance to NNRTI.

Finally, we analysed the demographic and clinical variables in the set of CRF19_cpx patients in our study. These characteristics were similar to those found for non-B infected patients in the Spanish AIDS Research Network Cohort (CoRIS) [25], except for the origin and risk category, since we mainly detected CRF19_cpx in Spanish MSM, while other non-B subtypes affect mainly heterosexual and immigrant patients. Regarding clinical and virological data, we can find no information supporting a greater pathogenicity of the CRF19_cpx recombinant, as seen in previous studies [7, 9]. Thus, few cases of AIDS were reported. The viral load and CD4 count at HIV-1 diagnosis were also similar to the non-B patients from CoRIS and even to the overall cohort, including all the subtype B patients [25–26]. All the patients were treated with first-line ART, with no case of treatment or virological failure reported. Consequently, the subtype CRF19_cpx does not seem to be associated with a superior pathogenicity in our cohort. Nevertheless, the status of outbreak in our area, mostly affecting MSM, means we cannot state that the characteristics in this case are specific for the CRF19_cpx subtype nor can they be generalized to other at-risk populations.

In summary, the CRF19_cpx recombinant has been mainly transmitted as an outbreak over a period of 6 years in southern Spain. Local young MSM are the major risk group affected. This subtype is associated with a high prevalence of cross-class primary resistance to NNRTI in our area, with more than half the cases presenting the G190A resistance mutation. Unlike previous studies, the CRF19_cpx recombinant from Malaga seems less pathogenic, with few cases of AIDS and excellent response to first-line ART.

## Supporting information

S1 Fig Maximum likelihood fits of 24 different nucleotide substitution models. The best substitution model was chosen according to the lowest AIC (Akaike Information Criterion) score as the selection criterion.
(TIF)

S2 Fig Detailed view of Bayesian phylogenetic inference obtained by MrBayes v3.2 program. Reference sequences appear with their corresponding accession numbers while each study patient is represented in red by their sample ID. Numbers near each node show posterior probabilities.
(PDF)

S3 Fig Phylogenetic inference based on the partial *pol* gene obtained by the maximum likelihood method with bootstrap values as a measure of branch supports. **(A)** Phylogenetic relationship of our CRF19_cpx sequences with regards to another 254 reference sequences from the same subtype retrieved from LANL. Each patient is represented in red by their sample ID while reference sequences appear with their corresponding accession numbers. **(B)** Subtree with the clustering of patients within the outbreak, highlighting in dark grey shading the presence of the G190A mutation as applicable. Asterisks indicate the detection of V179I/A. Only bootstrap proportions ≥50% are shown.
(TIF)

S4 Fig ML tree inference based on the partial *pol* gene obtained by PHYML v.3.0 program and with SH-aLRT test as branch support. **(A)** Phylogenetic relationship of our CRF19_cpx sequences with another 254 reference sequences from the same variant retrieved from LANL. Each patient is represented in red by their sample ID while reference sequences appear with their corresponding accession numbers. **(B)** Subtree with the clustering of patients within the outbreak, highlighting in dark grey shading the presence of the G190A mutation as applicable. Asterisks indicate the detection of V179I/A. Only SH-aLRT values ≥80% are depicted.
(TIF)

S1 Table REGA assignment of the sequences included in this study. (DOC)

S2 Table Comparison of the four demographic models considered in the coalescent analysis of the HIV-1 CRF19_cpx outbreak, with their corresponding Akaike’s Information Criterion (AICM) values. tMRCA median and range dates (95% HPD) for each one is depicted.
(DOCX)

## References

[1] Casado G, Thomson MM, Sierra M, Nájera R. Identification of a novel HIV-1 circulating ADG intersubtype recombinant form (CRF19_cpx) in Cuba. J. Acquir. Immune Defic.Syndr. 2005; 40: 532–37. 16284528 10.1097/01.qai.0000186363.27587.c0

[2] Delatorre E, Bello G. Phylodynamics of the HIV-1 epidemic in Cuba. PLoS One. 2013; 8: e72448 doi: 10.1371/journal.pone.0072448 24039765 10.1371/journal.pone.0072448 PMC3767668

[3] Blanco M, Machado LY, Díaz H, Ruiz N, Romay D, Silva E. HIV-1 Genetic variability in Cuba and implications for transmission and clinical progression. MEDICC Rev. 2015; 17: 25–31. 26947278 10.37757/MR2015.V17.N4.6

[4] Pérez L, Thomson MM, Bleda MJ, Aragonés C, González Z, Pérez J, et al HIV Type 1 molecular epidemiology in Cuba: high genetic diversity, frequent mosaicism, and recent expansion of BG intersubtype recombinant forms. AIDS Res Hum Retroviruses. 2006; 22: 724–33. doi: 10.1089/aid.2006.22.724 16910827 10.1089/aid.2006.22.724

[5] Pérez L, Kourí V, Alemán Y, Abrahantes Y, Correa C, Aragonés C, et al Antiretroviral drug resistance in HIV-1 therapy-naive patients in Cuba. Infect Genet Evol. 2013; 16: 144–50. doi: 10.1016/j.meegid.2013.02.002 23416260 10.1016/j.meegid.2013.02.002

[6] Machado LY, Blanco M, Dubed M, Díaz HM, Ruiz NM, Váldes N, et al HIV type 1 genetic diversity in newly diagnosed Cuban patients. AIDS Res Hum Retroviruses. 2012; 28: 956–60. doi: 10.1089/AID.2011.0295 22059433 10.1089/aid.2011.0295

[7] Kouri V, Khouri R, Alemán Y, Abrahantes Y, Vercauteren J, Pineda-Peña AC, et al CRF19_cpx is an evolutionary fit HIV-1 variant strongly associated with rapid progression to AIDS in Cuba. EBioMedicine. 2015; 2: 244–54. doi: 10.1016/j.ebiom.2015.01.015 26137563 10.1016/j.ebiom.2015.01.015 PMC4484819

[8] Machado LY, Dubed M, Díaz H, Ruiz N, Romay D, Váldes N, et al Transmitted HIV type 1 drug resistance in newly diagnosed Cuban patients. AIDS Res Hum Retroviruses. 2013; 29: 411–14. doi: 10.1089/AID.2012.0183 22985307 10.1089/AID.2012.0183

[9] Kouri V, Alemán Y, Pérez L, Pérez J, Fonseca C, Correa C, et al High frequency of antiviral drug resistance and non-B subtypes in HIV-1 patients failing antiviral therapy in Cuba. J Int AIDS Soc. 2014; 17 (4 Suppl 3): 19754 doi: 10.7448/IAS.17.4.19754 25397499 10.7448/IAS.17.4.19754 PMC4225368

[10] El Moussi A, Thomson MM, Delgado E, Cuevas MT, Nasr M, Abid S, et al Genetic diversity of HIV-1 in Tunisia. AIDS Res Hum Retroviruses. 2017; 33: 77–81. doi: 10.1089/AID.2016.0164 27473255 10.1089/AID.2016.0164

[11] Patiño Galindo JA, Torres-Puente M, Gimeno C, Ortega E, Navarro D, Galindo MJ, et al Expansion of the CRF19_cpx variant in Spain. J Clin Virol. 2015; 69: 146–49. doi: 10.1016/j.jcv.2015.06.094 26209397 10.1016/j.jcv.2015.06.094

[12] Thompson JD, Gibson TJ, Plewniak F, Jeanmougin F, Higgins DG. The Clustal X windows interface: flexible strategies for multiple sequence alignment aided by quality analysis tools. Nucleic Acids Research.1997; 25: 4876–82. 9396791 10.1093/nar/25.24.4876 PMC147148

[13] Guindon S, Dufayard JF, Lefort V, Anisimova M, Hordijk W, Gascuel O. New Algorithms and Methods to Estimate Maximum-Likelihood Phylogenies: Assessing the Performance of PhyML 3.0. Systematic Biology. 2010; 59: 307–21. doi: 10.1093/sysbio/syq010 20525638 10.1093/sysbio/syq010

[14] Shimodaira H, Hasegawa M. Multiple comparisons of log-likelihoods with applications to phylogenetic inference. Mol. Biol. Evol. 1999 16: 1114–16.

[15] Tamura K, Stecher G, Peterson D, Filipski A, Kumar S. MEGA6: Molecular Evolutionary Genetics Analysis Version 6.0. Mol Biol Evol. 2013; 30: 2725–29. doi: 10.1093/molbev/mst197 24132122 10.1093/molbev/mst197 PMC3840312

[16] Baele G, Lemey P, Bedford T, Rambaut A, Suchard MA, Alekseyenko AV. Improving the accuracy of demographic and molecular clock model comparison while accommodating phylogenetic uncertainty. Mol Biol Evol. 2012; 29: 2157–67. doi: 10.1093/molbev/mss084 22403239 10.1093/molbev/mss084 PMC3424409

[17] Ronquist F, Teslenko M, van der Mark P, Ayres DL, Darling A, Höhna S, et al MrBayes 3.2: efficient Bayesian phylogenetic inference and model choice across a large model space. Syst Biol. 2012; 61: 539–42. doi: 10.1093/sysbio/sys029 22357727 10.1093/sysbio/sys029 PMC3329765

[18] Bouckaert R, Heled J, Kühnert D, Vaughan T, Wu CH, Xie D, et al BEAST 2: a software platform for Bayesian evolutionary analysis. PLoS Comput Biol. 2014; 10: e1003537 doi: 10.1371/journal.pcbi.1003537 24722319 10.1371/journal.pcbi.1003537 PMC3985171

[19] Abecasis AB, Vandamme AM, Lemey P. Quantifying differences in the tempo of human immunodeficiency virus type 1 subtype evolution. J Virol. 2009; 83:12917–24. doi: 10.1128/JVI.01022-09 19793809 10.1128/JVI.01022-09 PMC2786833

[20] Hué S, Pillay D, Clewley JP, Pybus OG. Genetic analysis reveals the complex structure of HIV-1 transmission within defined risk groups. Proc Natl Acad Sci U S A. 2005; 102: 4425–29. doi: 10.1073/pnas.0407534102 15767575 10.1073/pnas.0407534102 PMC555492

[21] Liu TF, Shafer RW. Web resources for HIV type 1 genotypic-resistance test interpretation. Clin Infect Dis. 2006; 42: 1608–18. doi: 10.1086/503914 16652319 10.1086/503914 PMC2547473

[22] Pérez-Parra S, Alvarez M, Chueca N, García-Bujalance S, Pérez-Elías MJ, Molina JM, et al Características clínico-epidemiológicas, virológicas y filogenéticas de la variante CRF19_CPX en España. Enferm Infecc Microbiol Clin 2016; 34 (Suppl E3):76–77. Spanish.25533741

[23] Sena Corrales G, González Domenech CM, Viciana Ramos I, Palacios R, Mora Navas L, Clavijo Frutos E, et al Análisis de los subtipos de VIH-1 detectados en una cohorte de pacientes con infección por el VIH del área de Málaga. Enferm Infecc Microbiol Clin. 2017; 35 (Suppl E1): 130–131. Spanish.27296436

[24] Pérez L, Álvarez LP, Carmona R, Aragonés C, Delgado E, Thomson MM, et al Genotypic resistance to antiretroviral drugs in patients infected with several HIV type 1 genetic forms in Cuba. AIDS Res Hum Retroviruses. 2007; 23: 407–14. doi: 10.1089/aid.2006.0155 17411374 10.1089/aid.2006.0155

[25] Torrecilla García E, Yebra Sanz G, Llácer-Delicado T, Rubio García R, González-García J, García García F, et al Clinical, epidemiological and treatment failure data among HIV-1 non-B-infected patients in the Spanish AIDS Research Network Cohort. Enferm Infecc Microbiol Clin. 2016; 34: 353–60. doi: 10.1016/j.eimc.2015.07.016 26364856 10.1016/j.eimc.2015.07.016

[26] Sobrino-Vegas P, Gutiérrez F, Berenguer J, Labarga P, García F, Alejos-Ferreras B, et al The Cohort of the Spanish HIV Research Network (CoRIS) and its associated biobank; organizational issues, main findings and losses to follow-up. Enferm Infecc Microbiol Clin. 2011; 29: 645–53. doi: 10.1016/j.eimc.2011.06.002 21820763 10.1016/j.eimc.2011.06.002

