# Supplementary figures and images for: Emergence as an outbreak of the HIV-1 CRF19_cpx variant in treatment-naïve patients in southern Spain
Source: PLoS One. 2018 Jan 8;13(1):e0190544. doi: 10.1371/journal.pone.0190544 (PMC5757947; doi:10.1371/journal.pone.0190544)

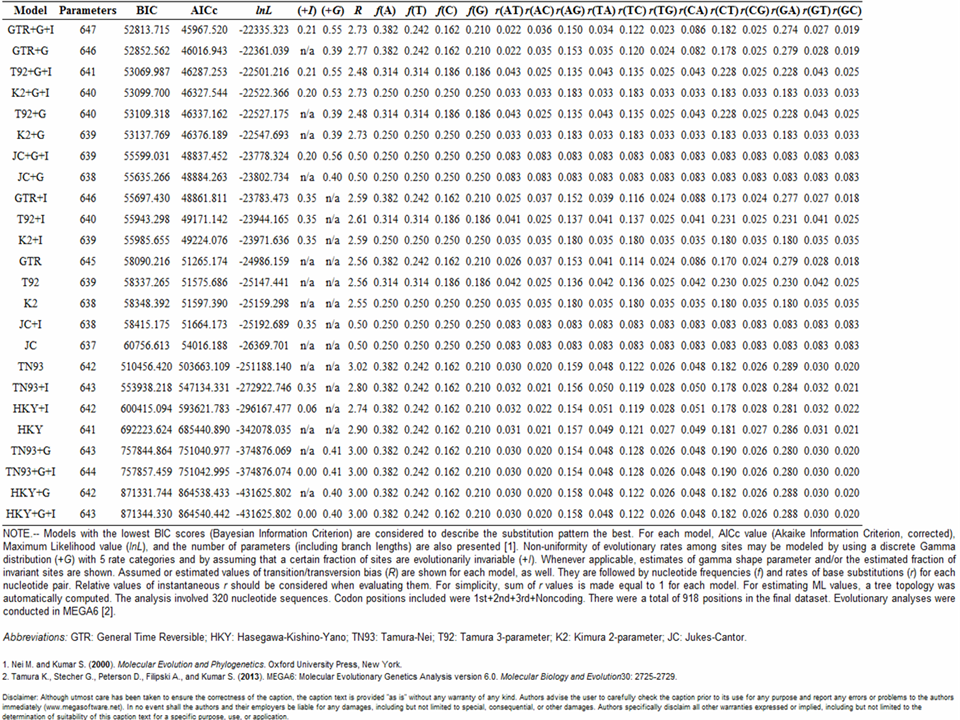

Supplement: S1 Fig — The best substitution model was chosen according to the lowest AIC (Akaike Information Criterion) score as the selection criterion. (TIF) [file pone.0190544.s001.tif]

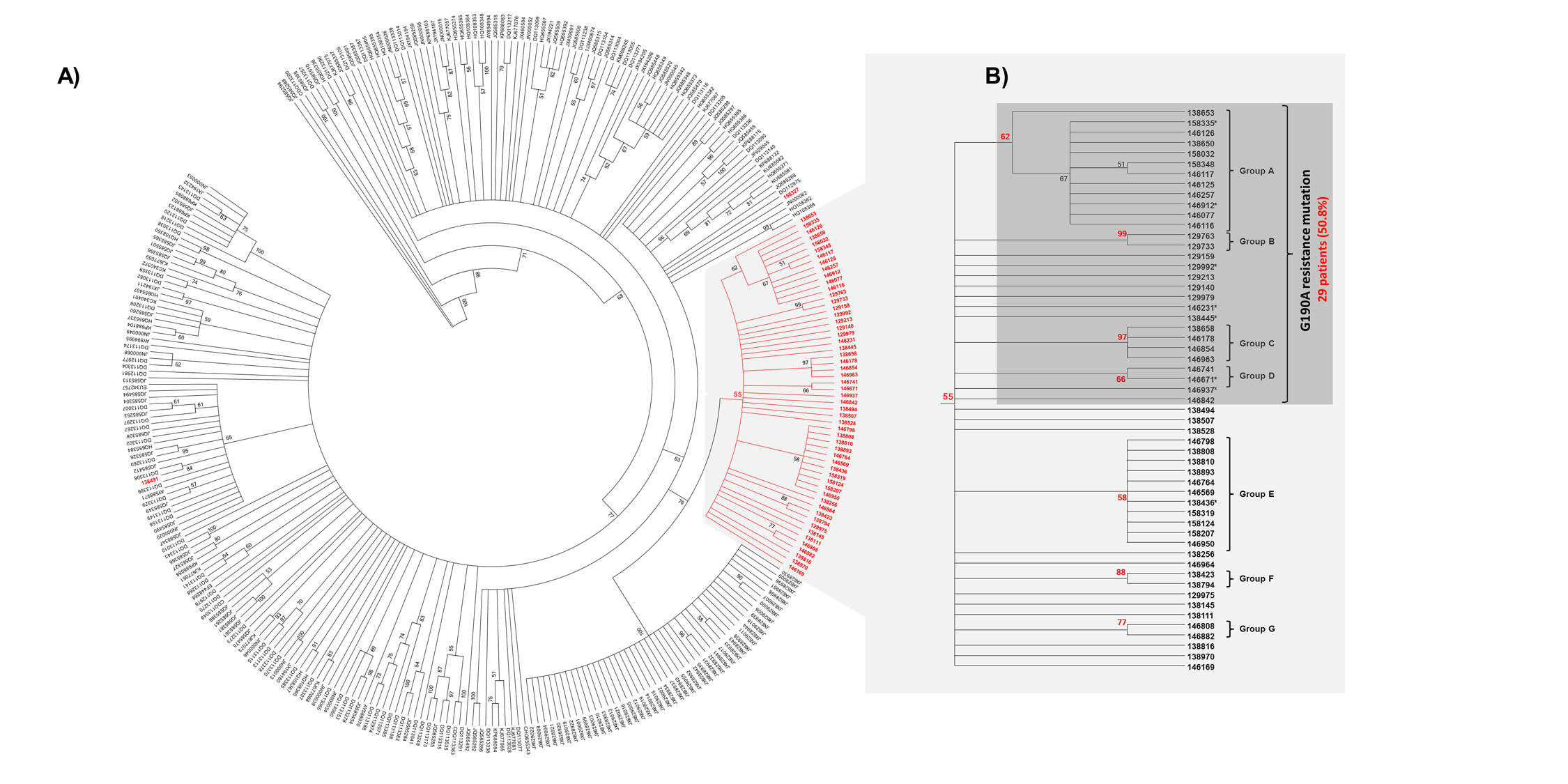

Supplement: S3 Fig — (A) Phylogenetic relationship of our CRF19_cpx sequences with regards to another 254 reference sequences from the same subtype retrieved from LANL. Each patient is represented in red by their sample ID while reference sequences appear with their corresponding accession numbers. (B) Subtree with the clustering of patients within the outbreak, highlighting in dark grey shading the presence of the G190A mutation as applicable. Asterisks indicate the detection of V179I/A. Only bootstrap proportions ≥50% are shown. (TIF) [file pone.0190544.s003.tif]

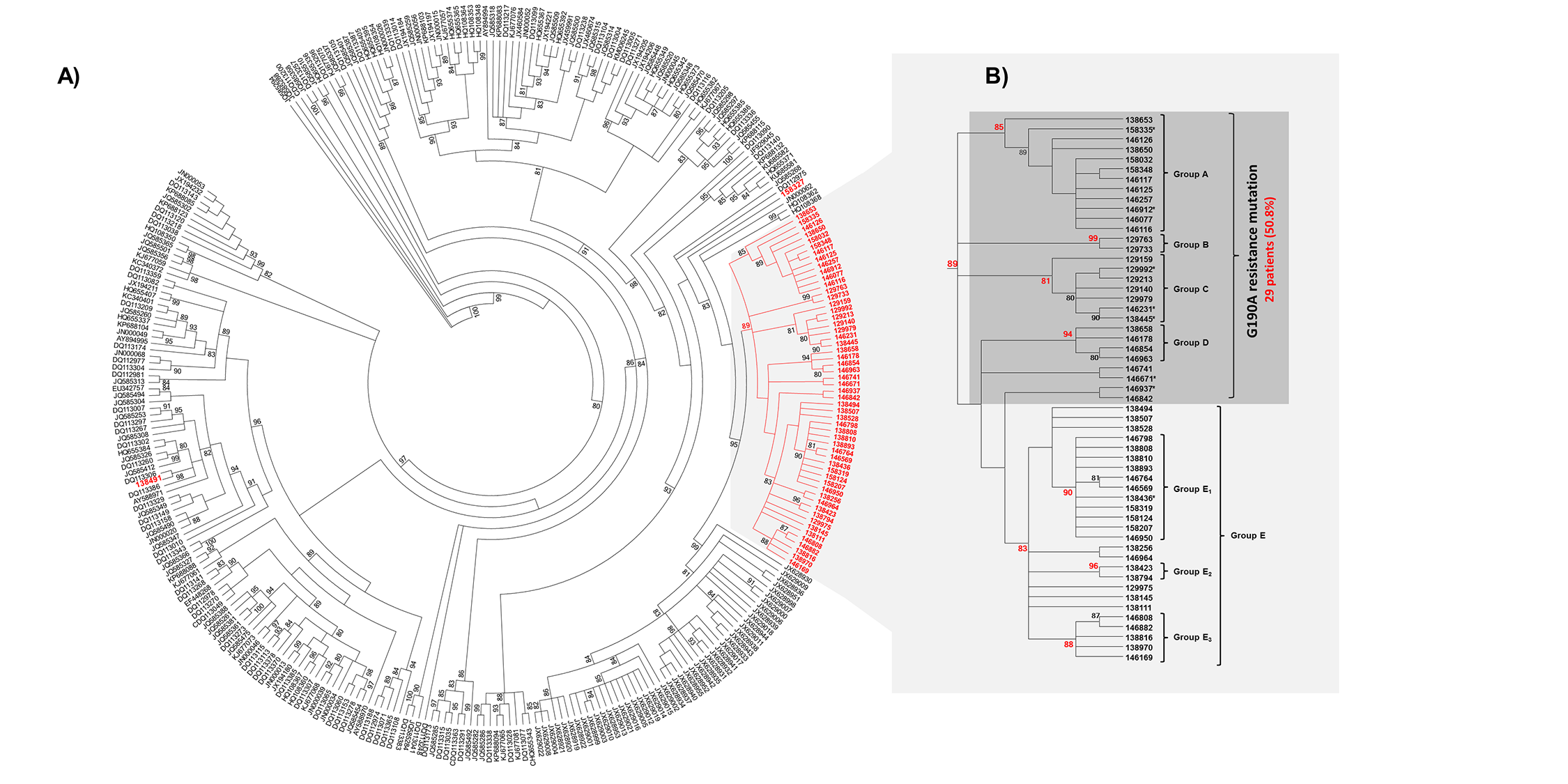

Supplement: S4 Fig — (A) Phylogenetic relationship of our CRF19_cpx sequences with another 254 reference sequences from the same variant retrieved from LANL. Each patient is represented in red by their sample ID while reference sequences appear with their corresponding accession numbers. (B) Subtree with the clustering of patients within the outbreak, highlighting in dark grey shading the presence of the G190A mutation as applicable. Asterisks indicate the detection of V179I/A. Only SH-aLRT values ≥80% are depicted. (TIF) [file pone.0190544.s004.tif]
