# Supplementary material for: Emergence as an outbreak of the HIV-1 CRF19_cpx variant in treatment-naïve patients in southern Spain
Source: PLoS One. 2018 Jan 8;13(1):e0190544. doi: 10.1371/journal.pone.0190544 (PMC5757947; doi:10.1371/journal.pone.0190544)
Supplement: S1 Table — (DOC) [file pone.0190544.s005.doc]

**S1 Table. REGA assignment of the sequences included in this study.**

| **REGA Assignment** | **Number of sequences** | **Percentage** |
| --- | --- | --- |
| **HIV-1 CRF19_cpx** | 50 | 87.7% |
| **Recombinant of 19_cpx, B** | 4 | 7.02% |
| **HIV-1 Subtype D (19_cpx)** | 3 | 5.26% |
| ***Total*** | 57 | 100% |
