# Supplementary material for: Emergence as an outbreak of the HIV-1 CRF19_cpx variant in treatment-naïve patients in southern Spain
Source: PLoS One. 2018 Jan 8;13(1):e0190544. doi: 10.1371/journal.pone.0190544 (PMC5757947; doi:10.1371/journal.pone.0190544)
Supplement: S2 Table — tMRCA median and range dates (95% HPD) for each one is depicted. (DOCX) [file pone.0190544.s006.docx]

**S2 Table. Comparison of the four demographic models considered in the coalescent analysis of the HIV-1 CRF19_cpx outbreak, with their corresponding Akaike’s Information Criterion (AICM) values. tMRCA median and range dates (95% HPD) for each one is depicted.**

| **Demographic model** | **AICM value(+/- SE)** | **tMRCA median** | **tMRCA range dates** |
| --- | --- | --- | --- |
| **Extended Bayesian Skyline** | 7059.009 (+/- 0.689) | 2009.9 | 2008.9-2010.8 |
| **Constant Population** | 7063.301 (+/- 0.685) | 2008.9 | 2007.6-2009.9 |
| **Exponential Population** | 7061.143 (+/- 0.525) | 2008.9 | 2007.8-2009.9 |
| **Bayesian Skyline** | 7058.99 (+/- 0.59) | 2009.0 | 2007.5-2010.0 |

AICM: Akaike information criterion; SE: Standard error.
