# Supplementary material for: Emergence as an outbreak of the HIV-1 CRF19_cpx variant in treatment-naïve patients in southern Spain
Source: PLoS One. 2018 Jan 8;13(1):e0190544. doi: 10.1371/journal.pone.0190544 (PMC5757947; doi:10.1371/journal.pone.0190544)

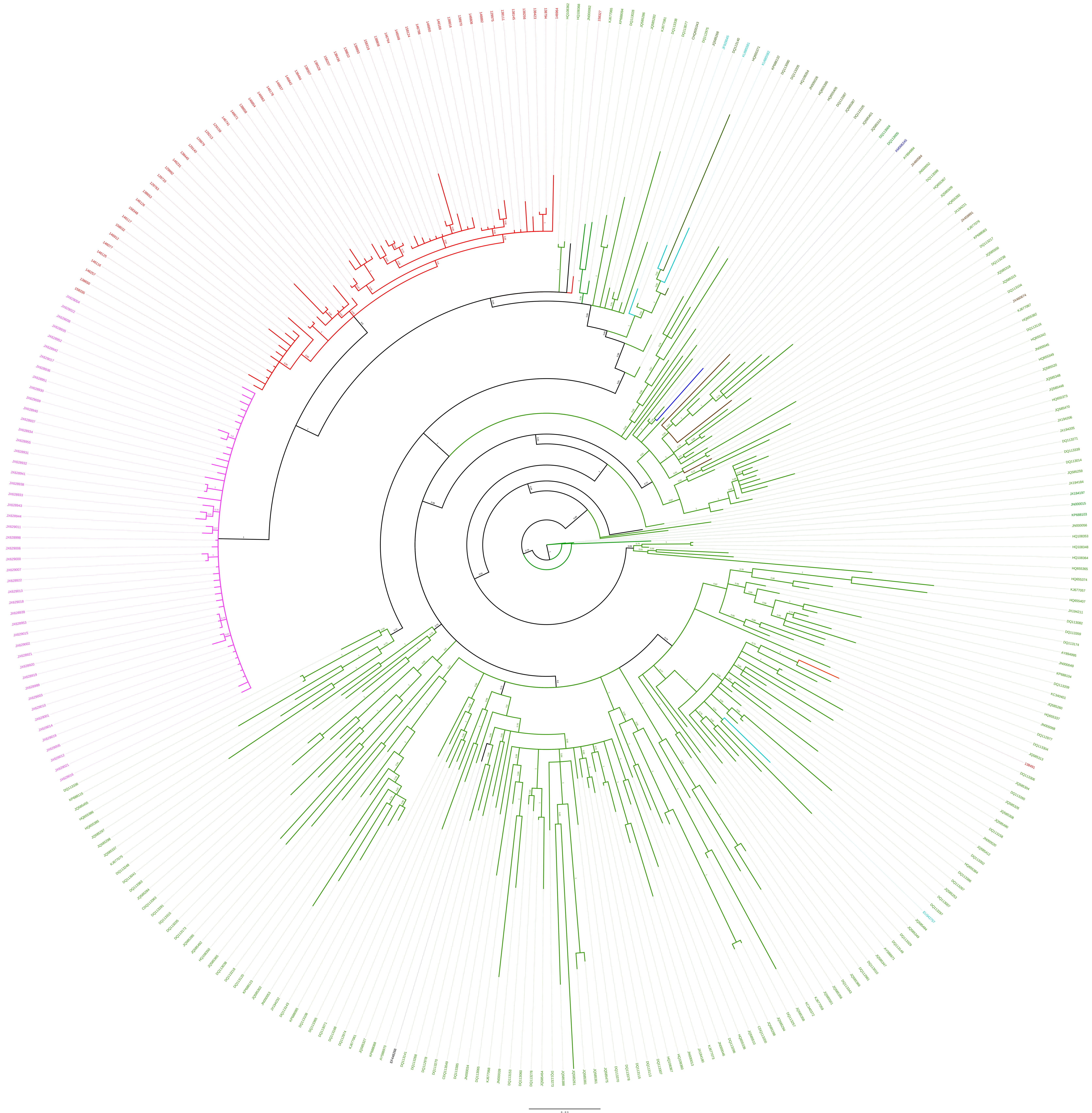

Supplement: S2 Fig — Reference sequences appear with their corresponding accession numbers while each study patient is represented in red by their sample ID. Numbers near each node show posterior probabilities. (PDF) [file pone.0190544.s002.pdf]
